# Axl-EGFR receptor tyrosine kinase hetero-interaction provides EGFR with access to pro-invasive signalling in cancer cells

**DOI:** 10.1038/oncsis.2016.66

**Published:** 2016-10-24

**Authors:** M Vouri, D R Croucher, S P Kennedy, Q An, G J Pilkington, S Hafizi

**Affiliations:** 1 Institute of Biomedical and Biomolecular Science, School of Pharmacy and Biomedical Sciences, University of Portsmouth, Portsmouth, UK; 2 Garvan Institute of Medical Research, Sydney, New South Wales, Australia; 3 St Vincent's Hospital Clinical School, University of New South Wales, Sydney, New South Wales, Australia; 4 School of Medicine and Medical Science, University College, Dublin, Ireland; 5 Systems Biology Ireland, University College Dublin, Belfield, Dublin 4, Ireland

## Abstract

Acquired resistance to conventional and targeted therapies is becoming a major hindrance in cancer management. It is increasingly clear that cancer cells are able to evolve and rewire canonical signalling pathways to their advantage, thus evading cell death and promoting cell invasion. The Axl receptor tyrosine kinase (RTK) has been shown to modulate acquired resistance to EGFR-targeted therapies in both breast and lung cancers. Glioblastoma multiforme (GBM) is a highly infiltrative and invasive form of brain tumour with little response to therapy. Both Axl and EGFR have been identified as major players in gliomagenesis and invasiveness. However, the mechanisms underlying a potential signalling crosstalk between EGFR and Axl RTKs are unknown. The purpose of this study was to investigate this novel and unconventional interaction among RTKs of different families in human GBM cells. With the use of western blotting, *in vitro* kinase activity, co-immunoprecipitation and bimolecular fluorescence complementation assays, we show that EGF stimulates activation of Axl kinase and that there is a hetero-interaction between the two RTKs. Through small interfering RNA knockdown and quantitative PCR screening, we identified distinct gene expression patterns in GBM cells that were specifically regulated by signalling from EGFR-EGFR, Axl–Axl and EGFR-Axl RTK parings. These included genes that promote invasion, which were activated only via the EGFR-Axl axis (*MMP9*), while EGFR-EGFR distinctly regulated the cell cycle and Axl–Axl regulated invasion. Our findings provide critical insights into the role of EGFR-Axl hetero-dimerisation in cancer cells and reveal regulation of cell invasion via Axl as a novel function of EGFR signalling.

## Introduction

The TAM (Tyro3, Axl and Mer) family of receptor tyrosine kinases (RTKs) share the structural feature of a two plus two combination of immunoglobulin-like domains and fibronectin type III repeats in the extracellular domain, followed by a transmembrane region and an intracellular tyrosine kinase domain. The natural ligands for the TAMs are the vitamin K-dependent proteins Gas6 (all three TAMs) and Protein S (Tyro3 and Mer only).^1, 2, 3^ The TAMs are well-known for a variety of regulatory roles. Axl is the most well studied TAM receptor to date, to which many cell biological roles have been attributed, including cell proliferation, survival, cell adhesion and migration,^4^ blood clot stabilisation, and regulation of inflammatory cytokines.^5, 6^ Moreover, Axl has been found to be overexpressed in a variety of cancers.^3, 7^ Even though Axl does not appear to be an initiator of the oncogenic process, its overexpression has been correlated with poor prognosis, promotion of increased invasiveness, epithelial-to-mesenchymal transition (EMT) and chemoresistance.^8, 9^

One study has shown that Axl overexpression in metastatic breast cancer is induced by the EMT mediators, Twist, Snail and Zeb2, in a process in which cells lose their adhesive properties and increase their migration potential.^9^ It was shown that Axl positively impacts on the expression of these mediators, and is possibly involved in a positive feedback loop.^9^ In a similar study, it was shown that Slug and H-Ras-induced EMT in basal-like breast cancer cells was dependent on the upregulation of the type III intermediate filament vimentin, which mediated invasion and migration in part through upregulating Axl.^10^ Axl signalling was also shown to be able to drive migration and extravasation of breast cancer cells from the bloodstream even in the absence of vimentin, indicating that Axl is a highly significant regulator of metastasis.^10^ This is further supported by the fact that Axl has been shown to regulate migration/invasion through induction of matrix metalloproteinase 9 (MMP9).^11^ In addition, Axl activation stimulated by its ligand Gas6 induced expression of the pro-survival family of proteins Bcl-2 and Bcl-xL and activated NF-κB, thus suppressing apoptosis.^12^ Axl has also been found to be overexpressed in castration-resistant prostate cancer and to significantly increase migration, invasion and proliferation via activation of PI3K and NF-κB pathways.^13^ Recent research also associated Axl overexpression with poor overall survival of glioblastoma multiforme (GBM) patients and increased glioma cell invasiveness.^14^ We recently showed that inhibition of Axl with a specific small molecule inhibitor hinders glioma cell survival, migration and invasion,^15^ thus highlighting the viability of targeting Axl specifically in cancer therapy.

In addition to its principal oncogenic role, numerous studies suggest that Axl overexpression mediates secondary resistance to both conventional and targeted therapies. The most noteworthy observation to date is acquired resistance to erlotinib in EGFR-mutated lung adenocarcinoma by induction of an EMT-like phenotype through Axl activation.^16^ Similarly, in another study, Axl overexpression correlated with acquired resistance to gefitinib.^17^ Recently, expression of Axl has been identified as a predictor of lack of response to the EGFR-targeted inhibitors lapatinib and erlotinib in triple-negative breast cancer cells.^18^ Interestingly, it has been found that EGFR signalling transactivates Axl and that this ligand-independent Axl activity, through possible hetero-interaction among different RTKs, could then diversify downstream signalling pathways beyond those triggered by EGFR alone. Activation of EGFR by EGF stimulation of MDA-MB-231 cells was shown to lead to both Axl and MET phosphorylation but not *vice versa*.^18^ EGFR-mediated Axl activation led to widespread downstream signalling changes that were blocked by Axl small interfering RNA, while Gas6 stimulation had lesser effects. This pivotal study therefore suggests that Axl can serve as an amplifier of EGFR signalling.^18^ Moreover, Axl overexpression lead to resistance to cetuximab in models of non-small cell lung cancer and head and neck squamous cell carcinoma.^19^ Interestingly, in the same study EGFR is shown to positively regulate Axl expression via the MAPK pathway in a positive feedback loop.^19^

In the present study, we have identified for the first time a protein-protein interaction between EGFR and Axl, in which Axl acts as a gateway for EGFR to access pro-invasive signalling pathways in GBM cells, which are known for their highly invasive and proliferative phenotype,^20^ and which overexpress both RTKs.^14, 21^ This unconventional hetero-interaction among RTKs of different classes diversifies the signalling pathways accessible to both RTKs, and indicates that EGFR-Axl signalling could act as a master regulator of chemoresistance in several cancers. Thus, it is of great importance to investigate the cancer phenotypic repercussions of such a partnership between these RTKs, in particular with regard to choice and timing of molecular targeted therapy.

## Results

### EGF activates Axl in SNB-19 cells via EGFR

**Figure 1:**
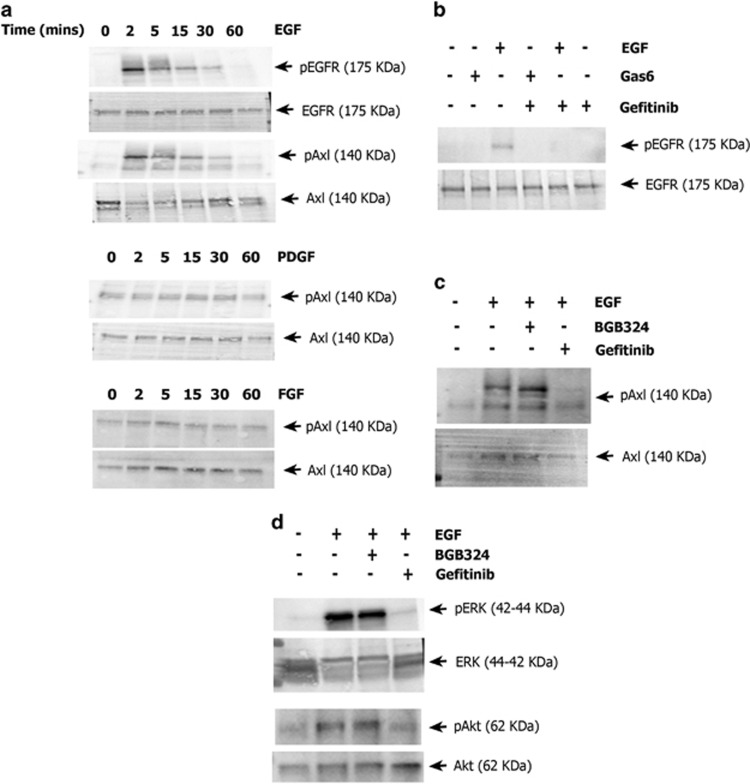
(**a**) Western blot of time course of EGFR phosphorylation by EGF (50 ng/ml) and of Axl phosphorylation by EGF (50 ng/ml), PDGF (10 ng/ml) and FGF (10 ng/ml). (**b**) Western blot of EGFR phosphorylation by EGF and Gas6 and its inhibition by gefitinib. (**c**) Western blot of Axl phosphorylation by EGF and its inhibition by gefitinib and BGB324. (**d**) Western blot of pERK and pAkt levels after EGF stimulation and influence of gefitinib and BGB324. Each blot is representative of three separate blots carried out for that experiment.

In order to investigate the relationship between EGFR and Axl in GBM cells, we first explored Axl activation (through residue-specific phosphorylation) in serum-starved SNB-19 cells in response to stimulation by the growth factors EGF, PDGF and FGF, over a time course of 0-60 min. EGF, in addition to causing a rapid increase in EGFR phosphorylation in under 2 min, also rapidly stimulated Axl phosphorylation within the same time period (Figure 1a). Interestingly, Axl activation by EGF was possible only with the fully glycosylated mature 140 kDa Axl but not the partially glycosylated Gas6 activated 120 kDa form (Figure 1a). The EGF-stimulated phosphorylation of Axl also occurred in a second GBM cell line but was not unique to cells of this cancer type, as it was also observed in cell lines derived from other solid cancers including breast and head and neck cancer (Supplementary Figure S1). In contrast, no significant change in Axl phosphorylation occurred after treatment with PDGF or FGF (Figure 1a).

To probe the specificity of the kinase-substrate relationship, pre-treatment with the highly selective Axl small molecule inhibitor BGB324^15, 22^ did not affect the Axl phosphorylation stimulated by EGF,^15^ whereas pre-treatment with the EGFR small molecule inhibitor gefitinib blocked Axl phosphorylation following EGF treatment (Figure 1c). Therefore, Axl activation in response to EGF is specific and relies on EGFR activity and not Axl activity, suggesting a direct phosphorylation of Axl by EGFR. To investigate whether this cross-phosphorylation observed in SNB-19 cells occurs in both directions, we tested EGFR phosphorylation following stimulation by Gas6, the natural ligand for Axl. Gas6 had no effect on EGFR phosphorylation (Figure 1b), indicating a unidirectional relationship between EGFR and Axl. In addition, western blotting of pERK and pAkt, two well-known downstream targets of RTK signalling pathways, showed no change in phosphorylation upon EGF stimulation in the presence of the Axl inhibitor BGB324, indicating that the two kinases are regulated predominantly by EGFR-EGFR or EGFR-Axl signalling in this context (Figure 1d).

### EGFR kinase directly activates Axl kinase *in vitro*

**Figure 2:**
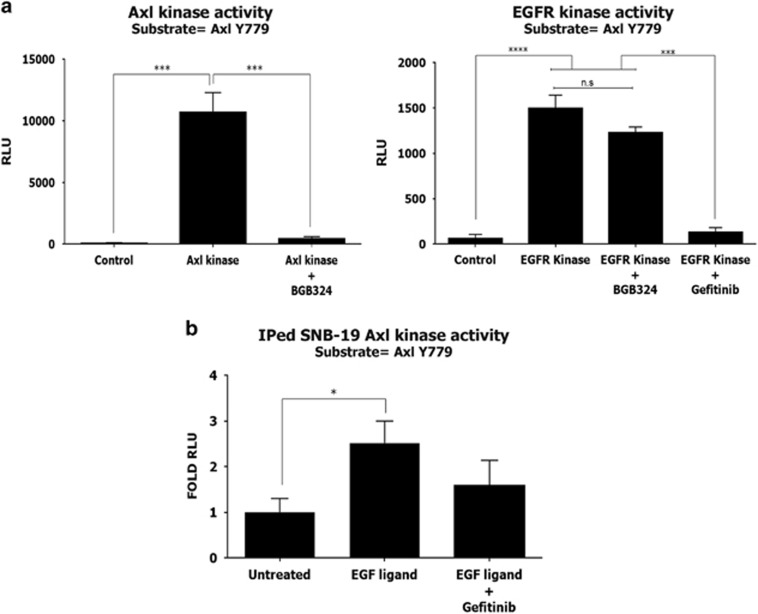
(**a**) *In vitro* kinase activity of recombinant Axl and EGFR kinases, with Axl Y779 peptide as substrate. Kinases were tested either alone or in the presence of the specific Axl or EGFR inhibitors, BGB324 (10μm) and gefitinib (10 μm), respectively. Values are normalised against background Y779 peptide luminescence. (**b**) *In vitro* kinase activity of native Axl immunoprecipitated from SNB-19 cells after EGF stimulation, with or without 1 h gefinitib (10 μm) pre-treatment. Data are mean±s.e.m. (*N*=3 separate experiments); ****P<0.0001, **P<0.01, *P<0.05 versus untreated.

Having observed that EGF stimulation leads to native Axl phosphorylation in living cells, we investigated the potential of EGFR to activate Axl in a cell-free *in vitro* system. Both recombinant active Axl and EGFR kinases were able to phosphorylate the Axl substrate peptide containing Y779, a residue that is a well-known docking site in Axl for multiple signalling proteins and a marker of Axl activation^23^ (Figure 2a). The *in vitro* phosphorylation of Axl Y779 by recombinant Axl and EGFR kinases was also inhibited by their respective small molecule inhibitors BGB324 and gefinitib, whereas BGB324 had no effect on EGFR kinase activity (Figure 2a).

In addition, we also determined the influence of EGF-EGFR on native Axl kinase activity in cells. Axl immunoprecipitated (IPed) from SNB-19 cells following 5 min incubation with EGF, exhibited a greater *in vitro* activity through phosphorylation of the Axl Y779 peptide (Figure 2b). Therefore, Axl is a direct substrate of EGFR kinase *in vitro*, as well as *in vivo* in intact cells following activation of EGFR by EGF.

### Physical association of EGFR and Axl in SNB-19 cells

**Figure 3:**
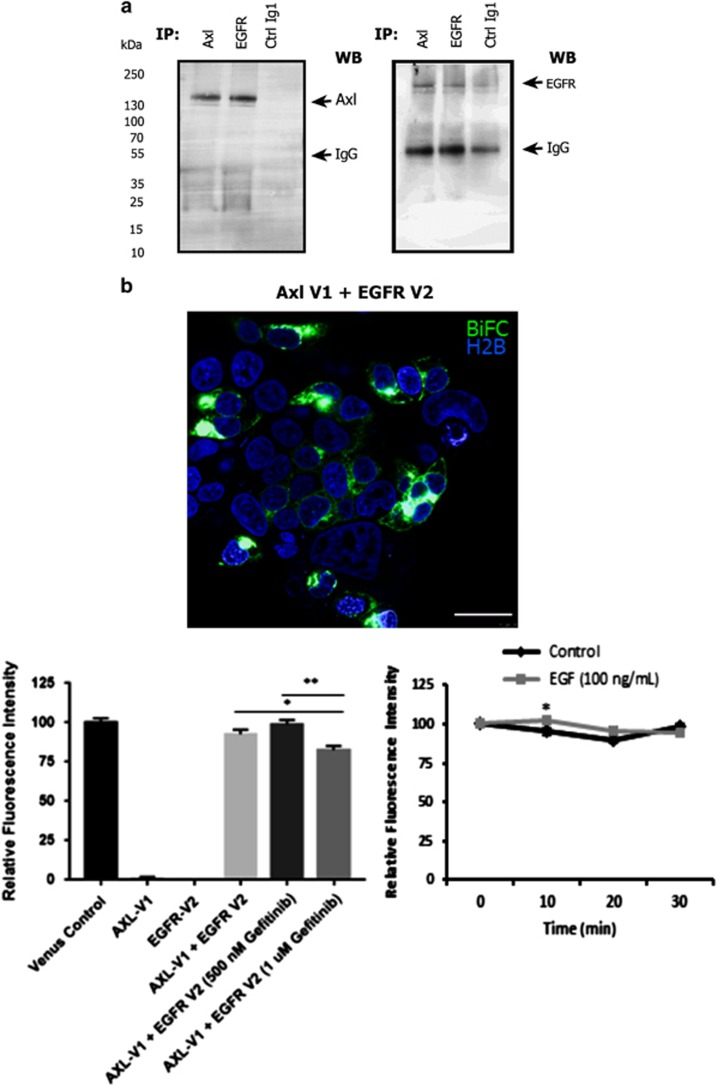
Hetero-interaction of EGFR and Axl RTKs. (**a**) CoIP of Axl and EGFR followed by western blot probing for presence in the complexes of Axl (left blot) and EGFR (right blot). Control coIP antibody was anti-GAPDH (Ctrl Ig1). (**b**) Bimolecular fluorescence complementation (BiFC) images showing EGFR-Axl complex formation followed by fluorescence quantitation bar graph and fluorescence intensity over time following EGF addition. Data are mean±s.e.m. (*N*=3 separate experiments); **P<0.01, *P<0.05 versus untreated.

Next, we investigated whether the observed unidirectional transactivation of Axl by EGFR occurs through a physical interaction between the two RTKs. EGFR coIPed with Axl even in the absence of EGFR stimulation by EGF (Figure 3a), indicating the existence of an EGFR-Axl complex present at the cell membrane. Using bimolecular fluorescence complementation imaging, we observed a constitutive association between Axl and EGFR proteins at the cell membrane regardless of EGF presence (Figure 3b). Inhibition of EGFR with gefinitib had only slight effects on the formation of the complex, with a small significant decrease in fluorescence following inhibitor treatment.

### EGFR induces upregulation of pro-invasive genes via Axl

**Figure 4:**
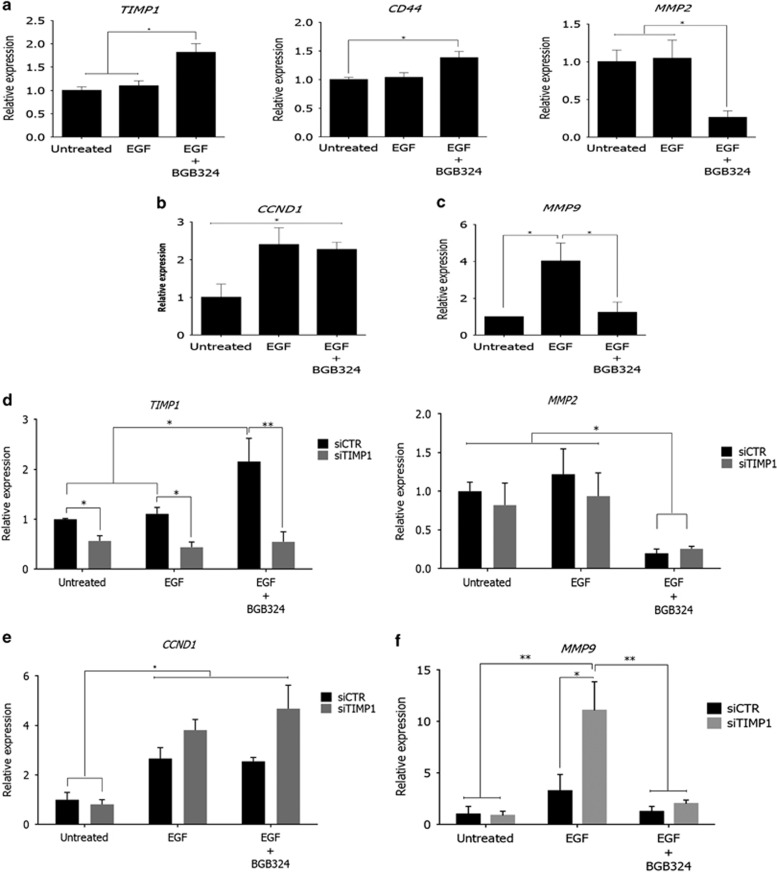
EGFR-Axl hetero-interaction regulates the balance between gene expression for invasive and proliferative signalling in SNB-19 cells. qRT-PCR analysis of (**a**) *TIMP1*, *MMP2, CD44*, (**b**) *CCND1* and (**c**) *MMP9* genes in SNB-19 cells treated for 24 h with 50 ng/ml EGF with or without 2 μM BGB324. Also, qRT-PCR analysis of (**d**) *TIMP1*, *MMP2*, (**e**) *CCND1* and (**f**) *MMP9* genes in SNB-19 cells with small interfering RNA knockdown of TIMP1 over 3 days, in the presence of EGF (50 ng/ml) with or without Axl blockade by BGB324 (2 μm). Data are mean±s.e.m. (*N*=3 independent experiments); ****P<0.0001, ***P<0.001, **P<0.01, * *P*<0.05.

Axl has been extensively linked to increased chemoresistance by activating EMT in a variety of cancers. To investigate any crosstalk between Axl and EGFR signalling in SNB-19 cells, we treated cells with EGF with or without concomitant blockade of Axl–Axl homodimer activation with BGB324, thus directing the effect of EGF towards the EGFR homodimers or Axl-EGFR heterodimers only. These treatments were then followed by qRT-PCR analysis of the expression of 15 EMT-related genes (*CD44, CD24, CD133, ALDH1, CCND1, CDK4, KRT19, NOTCH1, TWIST, MMP9, AKT2, TIMP1, AKT1, MMP2* and *PI3K)*. Out of these, we observed a significant change in the mRNA levels of *CCND1, TIMP1*, *CD44, MMP2* and *MMP9* genes (Figures 4a and c, respectively). *TIMP1* and *CD44* mRNA levels were significantly increased (Figure 4a) when Axl homodimer was blocked during EGF stimulation, whereas *CCND1* mRNA was significantly increased by EGF stimulation irrespective of Axl–Axl kinase activity (Figure 4b). Interestingly, EGF stimulation increased *MMP9* mRNA levels, while concomitant Axl inhibition by BGB324 blocked this, despite presence of EGF (Figure 4c). Also, in contrast, *MMP2* was significantly reduced by EGF stimulation only when Axl signalling was inhibited (Figure 4a).

### EGFR positively regulates cell invasion signalling via Axl

**Figure 5:**
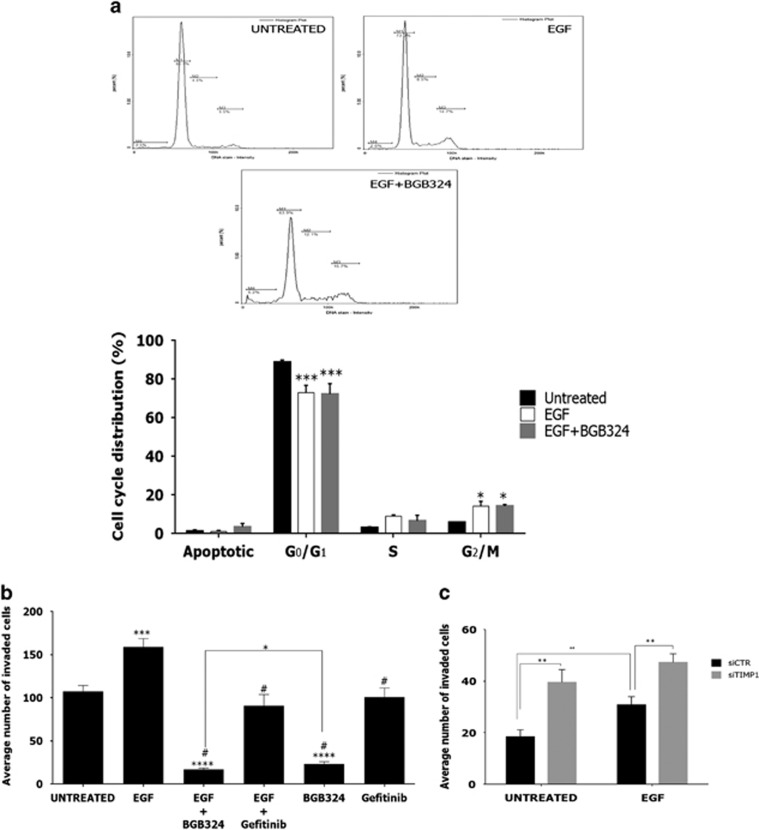
(**a**) Cell cycle analysis of cells treated for 24 h with EGF alone or EGF in combination with BGB324. (**b**) Invasion assay for SNB-19 cells following treatment with EGF, BGB324 and gefinitib using PDGF-AA as chemoattractant. (**c**) Invasion assay for SNB-19 cells with small interfering RNA knockdown of TIMP1 over 3 days, in the presence of EGF (50 ng/ml). Data are mean±s.e.m. (*N*=3 independent experiments); ****P<0.0001, ***P<0.001, **P<0.01, *P<0.05 compared to either untreated/controls or as indicated. ^#^ indicates significance when compared with EGF treatment.

We have previously shown Axl signalling to regulate the migration and invasion of the GBM cells used in this study.^15^ Therefore, identifying genes involved in invasion to be altered by EGFR-Axl signalling was of particular interest. In order to functionally evaluate the importance of this novel interaction, we treated cells with EGF with or without Axl kinase blockade by BGB324 or EGFR by gefitinib and subsequently observed its effect on GBM cell invasion (Figure 5b), in the absence and presence of the specific RTK inhibitors. EGF stimulation significantly increased cell invasion, an effect that was counteracted by Axl blockade. Most importantly, combination of EGFR stimulation with Axl–Axl inhibition led to a greater decrease in invasion compared to Axl–Axl inhibition alone. Moreover, gefitinib was able to reduce cell invasion back to basal levels. Knockdown of TIMP1 by small interfering RNA resulted in increased invasion regardless of EGFR–EGFR or EGFR–Axl stimulation/inhibition, indicating that they act via TIMP1 to regulate invasion (Figure 5c). In order to further probe the mechanisms behind this behaviour, we monitored the mRNA levels of the other altered genes under these conditions and upon TIMP1 knockdown. We found that *MMP9* mRNA levels were increased upon EGF stimulation, an effect that was enhanced by TIMP1 knockdown (Figure 4f). Interestingly, Axl kinase inhibition blocked the EGF-induced upregulation of *MMP9*, irrespective of TIMP1 knockdown. In contrast to *MMP9*, TIMP1 knockdown had no effect on the EGF-induced changes in *CD44* and *MMP2* expression (Figure 4d). Therefore, these results indicate that Axl positively regulates cancer cell invasion via the TIMP1-MMP9 axis, also that EGF activates this pathway only via Axl, and moreover that discrete EGFR signalling alone in fact directly counteracts Axl invasive signalling.

### EGFR stimulates cell cycle progression independent of Axl

We further investigated the observed increase in the *CCND1* gene, which codes for cyclin D1, on the cell cycle in SNB-19 cells. Cell cycle analysis after treatment with EGF alone or in combination with BGB324 revealed a decrease of cells in the G_1_ phase with a concomitant increase in the G_2_/M population, irrespective of Axl–Axl kinase activity (Figure 5a). This reflected the observed EGF-induced increase in *CCND1* mRNA expression (Figure 4b), occurring irrespective of TIMP1 expression (Figure 4e) as a confirmation of its separation from invasion signalling. Therefore, EGFR-EGFR signalling alone stimulates cell cycle progression in SNB-19 cells in addition to attenuating Axl invasive signalling.

## Discussion

In this study, we have identified for the first time a direct interaction between EGFR and Axl RTKs, with EGF/EGFR-induced activation of Axl as a novel signalling pathway to invasion in cancer cells. Our results here show that Axl heterodimerises with EGFR, as shown by co-IP and bimolecular fluorescence complementation imaging, which adds diversification to downstream signalling and subsequently cell behaviour. EGF, and not the other growth factors PDGF and FGF, stimulated activation of Axl directly through EGFR–Axl hetero-interaction. This occurred in a unidirectional manner, i.e. that EGFR activated Axl but not *vice versa*. The activation of Axl was shown to occur through direct phosphorylation by EGFR of Axl tyrosine 779, one of the key residues within Axl that serves as a multi-substrate docking site for further downstream signalling.^23^ The directionality of the relationship between EGFR and Axl was evident from use of specific inhibitors of the two RTKs. BGB324 inhibited intrinsic Axl kinase activity and therefore Axl–Axl, as has previously been reported by ourselves^15^ and others. However, BGB324 did not prevent EGFR from phosphorylating Axl and thereby creating docking sites in Axl (such as pY779) for intracellular signalling mediators; this scenario involves EGFR–Axl hetero-association. Therefore, a distinction is apparent between Axl–Axl signalling and EGFR–Axl signalling, in which Axl is the passive partner as a kinase.

In addition, EGFR stimulated activation of native Axl protein in SNB-19 cells, an effect that was reduced by specific inhibition of EGFR. However, it is possible that some of the Y779 phosphorylation measured in this kinase assay could be due to EGFR pulled down along with Axl, thus the kinase activity exhibited *in vitro* could be due to both RTKs. Nevertheless, our combined experiments show that EGFR enhances Axl activity, and in agreement with our findings, Meyer and colleagues have shown that EGF stimulation transactivated Axl in breast cancer cells (which we also observed here), which intensified downstream EGFR signalling.^18^ Tyrosine 779 of Axl is a major kinase target and docking site for several intracellular signalling adaptors and kinases.^1, 23^ Therefore, Axl phosphorylation by EGFR on key residues such as this would be expected to form new protein docking sites and subsequent activation of alternative downstream signalling pathways which would otherwise not be canonically regulated by either receptor alone through homo-dimerisation. Recently, Elkabets *et al.* showed that Axl activates the EGFR/PKC/mTOR axis in head and neck and oesophageal squamous cell carcinomas in order to arbitrate resistance to PI3Kα inhibitors.^24^ However, here we did not observe any effect on EGFR phosphorylation by Gas6-induced Axl activation (which we have previously shown in this cell line^15^). Furthermore, specific Axl kinase small molecule inhibition did not hinder its phosphorylation by EGFR,^15^ indicating that the event occurs independently of Axl kinase. The specificity of BGB324 at these concentrations for Axl versus Tyro3 and EGFR RTKs has been shown in previous work from our lab.^15^

We identified *MMP9* gene to be specifically regulated by Axl–EGFR signalling but neither RTK alone. Furthermore, the *MMP2* gene as well as the anti-invasive gene *TIMP1* seem to be exclusively regulated by Axl–Axl signalling as EGF stimulation had no effect on their expression, but Axl blockade resulted in a significant increase in *TIMP1* levels whereas *MMP2* levels where significantly reduced. On the other hand, the *CCND1* gene appears to be exclusively regulated by EGFR–EGFR signalling irrespective of Axl kinase activity; EGF stimulation significantly upregulated *CCDN1*, which was not altered by Axl kinase blockade.

In order to further investigate the effect of *TIMP1* signalling on cell behaviour, we performed cell invasion assays where we transiently knocked down TIMP1 and stimulated with EGF. As expected, *TIMP1* knockdown on its own resulted in a significant increase in cell invasion, while EGF stimulation significantly added to the *TIMP1* knockdown effect. This observation indicates a pro-invasive signalling outcome for the EGFR-Axl hetero-interaction, which is normally counteracted by EGFR stimulation of *TIMP1* expression. Moreover, EGF stimulation led to a significant increase in invasion, an effect that was counteracted by Axl–Axl kinase inhibition. However, EGFR activation in combination with Axl kinase inhibition significantly reduced baseline cell invasion to a greater extent than Axl inhibition alone. Indeed, an increase in *TIMP1* mRNA was observed upon EGF stimulation in concert with Axl inhibition, which can account for this phenomenon. Furthermore, EGFR inhibition alone was able to restore the number of invaded cells back to baseline indicating that EGFR alone cannot account for the invasive potential of these cells.

With the purpose of identifying why EGFR activation further increased cell invasion under these conditions, we also investigated and confirmed increases in *MMP2* and *MMP9* genes. Interestingly, previous studies have established *MMP9* to be directly regulated by Axl in breast cancer^11^ and *MMP2* in ovarian cancer^25^ independently of TIMP1 activity. Given our results, we found that in GBM cells, the EGFR-Axl axis promotes invasion through upregulation of *MMP9*, while Axl regulates *MMP2* expression independently of EGFR or TIMP1. Our results are supported by previous studies that reported that MMP9 binds to TIMP1 while failing to bind MMP2.^26^ Furthermore, Axl has been reported to regulate *MMP2* at the transcriptional level in ovarian cancer through the PI3K pathway.^25^ We have previously shown Axl to activate the PI3K pathway in glioma and Axl inhibition to suppress it,^15^ indicating that the same regulation exists in glioma. In addition, the gene for cyclin D1, *CCND1,* was upregulated upon EGF stimulation, irrespective of Axl kinase activity. Coupled with this, cell cycle analysis revealed that EGF stimulation, with or without Axl blockade, drove cells to enter mitosis. Therefore, these data show that EGF/EGFR-EGFR signalling promotes cell proliferation through upregulation of TIMP1 and cyclin D1,^27^ while EGFR can also exploit Axl signalling to promote cell invasion through downregulation of *TIMP1* and *CCND1* with concomitant *MMP9* upregulation.

**Figure 6:**
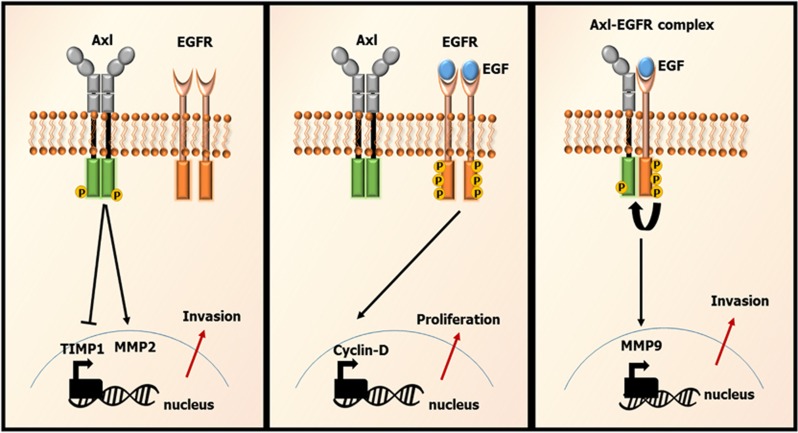
Schematic summary of the results of this study, showing Axl homo-dimerisation to inhibit *TIMP1* expression and promote *MMP2* expression (left panel), while EGFR homodimers promote *CCND1* expression (middle panel), and EGFR-Axl hetero-interaction, with unidirectional activation of Axl by EGFR, leads to activation of pro-invasive signalling pathways, involving *MMP9* as a central regulator (right panel).

Axl has previously been shown to mediate resistance to EGFR inhibitors by promoting EMT in breast^18^ and lung^16^ cancers. Therefore, based on this and our observations, it appears that EGFR and Axl can co-exist in local clusters on the plasma membrane, leading to subsequent activation-dependent enhancement of interactions after ligand stimulation. Therefore, it follows that drugs disrupting this complex interaction may be efficacious in counteracting such signal diversification and thereby more effective in combating both primary and secondary resistance in tumours with the appropriate molecular targets.^18^ In conclusion, we have shown a specific relationship between EGFR and Axl RTKs, enabled through a direct protein-protein interaction, which diversifies the signalling pathways available to EGFR. Having previously shown that Axl signalling is required for GBM cell invasion,^15^ we now show here for the first time that EGFR can also signal via Axl to promote cell invasion (Figure 6), a role for which EGFR, being a major mitogenic growth factor, is not well-known as a single entity. Furthermore, it is likely that EGFR-Axl signalling balances invasion and proliferation through the regulation of key genes such as *CCND1*, *TIMP1* and *CD44*; indeed, EGFR alone promotes expression of these genes while EGFR-Axl supresses their expression. Given these observations, it is conceivable that a combination of EGFR and Axl inhibition can be more effective than monotherapy for treating some cancer patients, in particular as a means to prevent or delay secondary resistance to targeted therapy.

## Materials and methods

### Cell lines and reagents

The immortalised human GBM cell line SNB-19 (DSMZ German cell culture bank) was authenticated and verified free of mycoplasma through in-house testing as previously described.^28^ Cells were cultured in Dulbecco's Modified Eagle Medium (Thermo Fisher Scientific, Loughborough, UK) supplemented with 10% foetal bovine serum (Lonza, Slough, UK), 1% l-glutamine (Life technologies, Paisley, UK) and 1% penicillin/streptomycin (Thermo Fisher Scientific). Cells were routinely grown in a humidified incubator with 5% CO_2_ at 37 °C, and subcultured through dissociation with trypsin/EDTA (Lonza) and proportional reseeding. Cells were always grown to near confluence (90%) before experimental use.

### Cell treatments

SNB-19 cells were serum starved for 24 h before being treated at indicated times with recombinant human proteins, including Gas6 (400 ng/ml), epidermal growth factor (EGF; 50 ng/ml), platelet-derived growth factor (PDGF; 10 ng/ml) or fibroblast growth factor (FGF; 10 ng/ml; Bio-techne, Abingdon, UK). In inhibition experiments, the EGFR small molecule inhibitor gefitinib (Santa Cruz Biotechnology, Santa Cruz, CA, USA) or the Axl small molecule inhibitor BGB324 (BerGenBio AS, Bergen, Norway) were pre-incubated with cells for 1 h prior to EGF stimulation.

### Western blotting

Cell extracts were separated by SDS-PAGE and proteins identified by western blotting, as previously described.^15^ Antibodies used for western blot detection were as follows: Axl (C-20), pEGFR, Akt 1/2/3 and pAkt 1/2/3 (Santa Cruz), EGFR (Cell Signalling Technology, Leiden, The Netherlands) and pAxl 779 (Bio-techne). Secondary antibodies used were horseradish peroxidase (HRP)-conjugated anti-rabbit (Dako, Cambridge, UK), anti-goat and anti-mouse Igs (Promega, Southampton, UK).

### Immunoprecipitation

SNB-19 cells were grown to confluency and treated with EGF (50 ng/ml) for 5 min prior to lysis. To extract proteins, cells were washed with ice-cold PBS and lysed in IP lysis buffer (Thermo Fisher). Following a 10 min centrifugation at 10 000 *g*, the lysates were pre-cleared by incubation with 10 μl protein A/G-agarose beads (Santa Cruz) for 1 h at 4 °C with constant rotation. Lysates were then incubated with 2 μg Axl or EGFR IP antibodies for 2 h at room temperature, with constant rotation. Antibodies against GAPDH or Tensin2 (Santa Cruz) were used as species-aligned negative control IP antibodies. Following incubation, 20 μl of protein A/G-agarose beads was added to each sample and incubated further for 2 h. The beads were then separated by centrifugation at 8000 *g* for 1 min and washed once with ice-cold IP lysis buffer and thrice with PBS (Thermo Fisher Scientific). Finally, 4 × SDS-PAGE loading buffer was added to the beads and solubilised samples underwent SDS-PAGE and western blotting.

### *In vitro* kinase activity assay

The Axl Kinase Enzyme System (Promega) was employed according to the manufacturer's protocol with minor modifications. Briefly, each of recombinant Axl or EGFR kinases (Stratech Scientific, Suffolk, UK) were incubated for 30 min with the substrate peptide (DCLDGLYALMSRC, 0.2 μg/μl; Pepceuticals Ltd., Leicester, UK) that contained tyrosine 779 (Y779) of human Axl within it, in the presence or absence of the EGFR inhibitor gefitinib (10 μm) or Axl inhibitor BGB324 (10 μM), in Kinase assay reaction buffer containing ATP (25 μm). DMSO was used as vehicle control instead of inhibitor. ADP-Glo reagent was then added to the wells and incubated at room temperature for 60 min followed by addition of Kinase detection reagent, with further incubation for 30 min at room temperature. Luminescence was read using a microplate luminometer (BMG Labtech Fluorstar Optima, Offenburg, Germany).

To determine the kinase activity of native Axl pulled down from SNB-19 cells by IP, the pellet of protein A/G-agarose beads obtained from Axl IP (described above) was washed a final time with Axl reaction buffer (8000 × *g* for 1 min). The Kinase assay reaction buffer, containing ATP (25 μM) and the Axl substrate peptide (0.2 μg/μl) (Promega) were added directly to the pellet, and the kinase reaction incubated for 30 min at room temperature. The reaction was stopped, developed and detected in the same way as for recombinant kinases above.

### Bimolecular fluorescence complementation

Bimolecular fluorescence complementation vectors were generated by gateway cloning with donor vectors containing Axl (pDONR223-Axl, Addgene plasmid 23945) or EGFR (pDONR223-EGFR, Addgene plasmid 23935).^29^ These were cloned into pDEST-ORF-V1 (Addgene plasmid 73637) and pDEST-ORF-V2 (Addgene plasmid 73638), respectively. An expression vector encoding full length Venus fluorescent protein was also utilised as control. For confocal microscopy, HEK-293T cells expressing an H2B-mCherry nuclear marker were grown on glass coverslips within a six-well plate. These were transfected with 500 ng of each vector (or Venus control) using Polyplus Jetprime and incubated for 16 h. The coverslips were prepared for confocal microscopy by fixation with 1% paraformaldehyde for 5 min at room temperature. For high-content analysis, HEK-293T cells expressing an H2B-mCherry nuclear marker were grown on Greiner CELLSTAR 96-well plates in phenol red free media (Thermo Fisher Scientific). Each well was transfected with 20 ng of each vector (or Venus control) using Polyplus Jetprime and incubated for 16 h, in the presence of absence of gefitinib at the concentrations indicated. Single cell fluorescence intensity was measured using the ArrayScan XTI Live High Content Platform (Thermo Fisher Scientific).

### Cell invasion assay

Invasion of SNB-19 cells through extracellular matrix was measured using a modified insert chamber coated with matrix proteins in 24-well culture plates, using PDGF-AA as chemoattractant, as previously described.^15^ Briefly, cells were treated with EGF (50 ng/ml), BGB324 (5 μm) and/or gefitinib (5 μm) for 16 h and allowed to invade through the pore-containing membrane over 16 h after which they were fixed with paraformaldehyde (Sigma, Poole, UK) and stained with 0.5% crystal violet (Fisher, Loughborough, UK) for 30 min. Invaded cells were counted in 5 separate fields. In some experiments, prior to the assay, cells were transfected with 10 nM TIMP1 or Control small interfering RNA (Santa Cruz) for 72 h, and/or treated with EGF (50 ng/ml) or BGB324 (2 μm) for 16 h.

### Quantitative reverse transcription polymerase chain reaction

Cellular total RNA was isolated using GeneJET RNA purification kit (Life Technologies) according to the manufacturer's protocol. First-strand cDNA was synthesised using a reverse transcription kit (nanoScript; PrimerDesign, Southampton, UK). Quantitative PCR (qPCR) amplification was performed in 96-well plates in a qPCR mastermix using either SYBR Green or fluorescent probes (Roche, Burgess Hill, UK) and run on a LightCycler 96 System (Roche). For the human EMT gene expression screen, the qPCR amplifications were performed using the Human CSC and EMT Biomarker RT Array Kit (NanoCinna Technologists Ltd., Tehran, Iran). Further qPCR investigations of individual genes were performed using pre-designed primers/probes for *TIMP1* (NM_003254), *MMP2* (NM_004530), *CCND1* (NM_053056), *CD44* (NM_001001389) (Integrated DNA Technologies; Leuven, Belgium). Relative expression analysis was performed using the equation *N*=N_0_ × 2^Cp^ (LightCycler 96 software; Roche), normalising against the gene for ATP synthase subunit beta (*ATP5B*) (Life Technologies).

### Cell cycle analysis

SNB-19 cells were serum starved for 24 h prior to being treated with 50 ng/ml EGF alone or in combination with 2.5 μm BGB324, or vehicle (DMSO) for 24 h. Following drug treatments, the cells were centrifuged at 400 *g* for 5 min, washed with PBS and resuspended in 70% ethanol/PBS (Thermo Fisher Scientific) for 2 h on ice. Following a PBS wash, the cells were incubated with 1 μg/ml DAPI, 0.1% Triton X-100 in PBS (Chemometec A/S, Allerød, Denmark) for 5 min at 37 °C. Thirty μl of stained cells was loaded onto a special chamber slide (NC-Slide A2) and cell populations were analysed for DAPI fluorescence (NucleoCounter NC-3000; Chemometec) according to the manufacturer's protocol.

### Statistical analyses

All data are expressed as mean±s.e.m., obtained from a minimum of 3 independent experiments, each constituting multiple replicates per condition as specified in the figure legends. Quantitative data were analysed by Analysis of Variance (ANOVA) with *post hoc* Tukey test for multiple comparisons with one control group or multiple time points per treatment, or paired *t*-test for pairwise comparisons of control with treatment. Statistical analyses of data and their graphical representations were performed using Prism software (GraphPad Software Inc, San Diego, CA, USA). The level of statistical significance is indicated in the figures and accompanying legends, with *P*<0.05 considered as statistically significant. Western blot image processing was performed using Adobe Photoshop CC 2014 software (Adobe Systems Incorporated, San Jose, CA, USA).
