# Supplementary material for: Axl-EGFR receptor tyrosine kinase hetero-interaction provides EGFR with access to pro-invasive signalling in cancer cells
Source: Oncogenesis. 2016 Oct 24;5(10):e266–. doi: 10.1038/oncsis.2016.66 (PMC5117851; doi:10.1038/oncsis.2016.66)
Supplement: Supplementary Figure S1 [file oncsis201666x1.docx]

**Vouri *et al*. - Supplementary Figure S1**


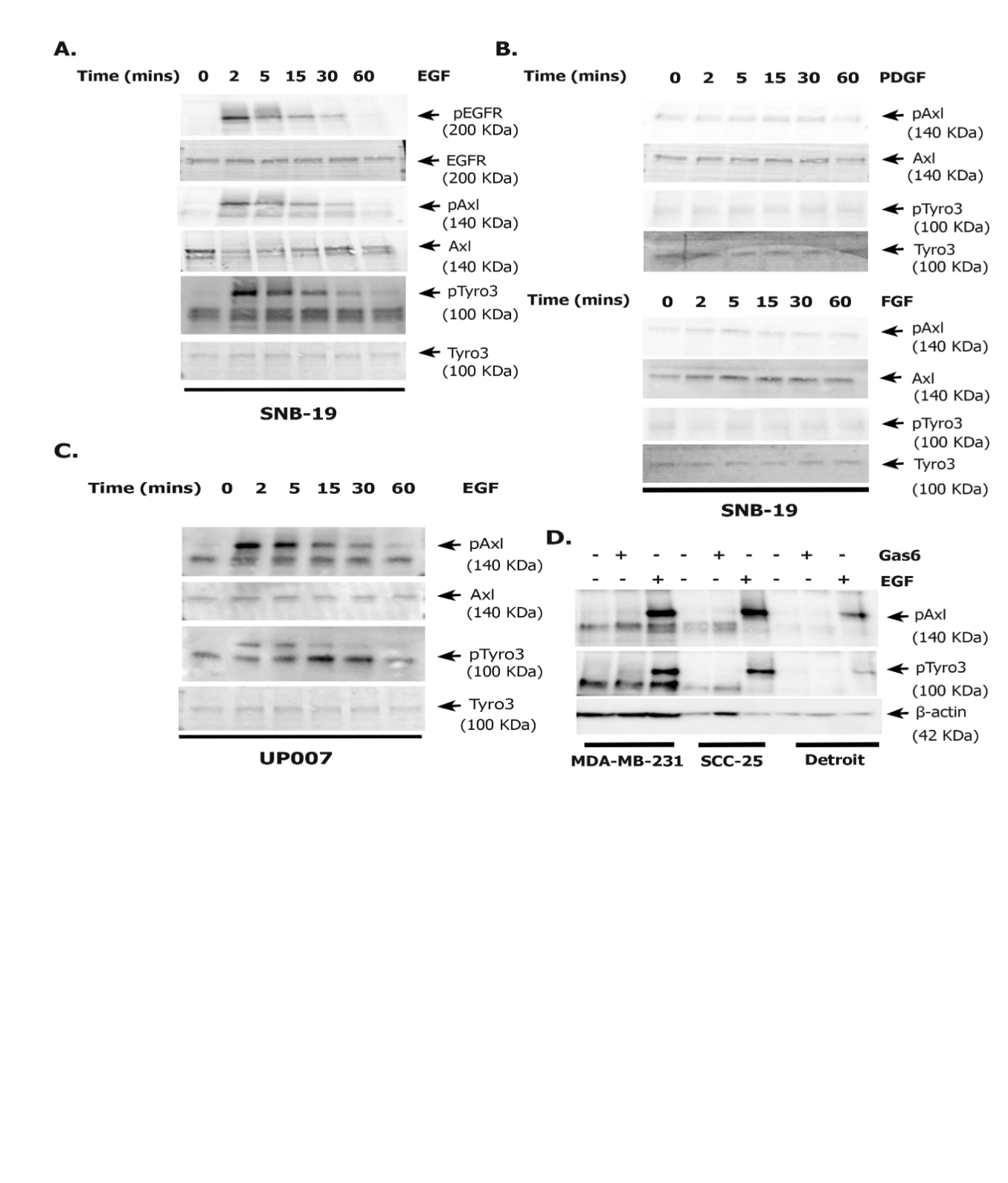

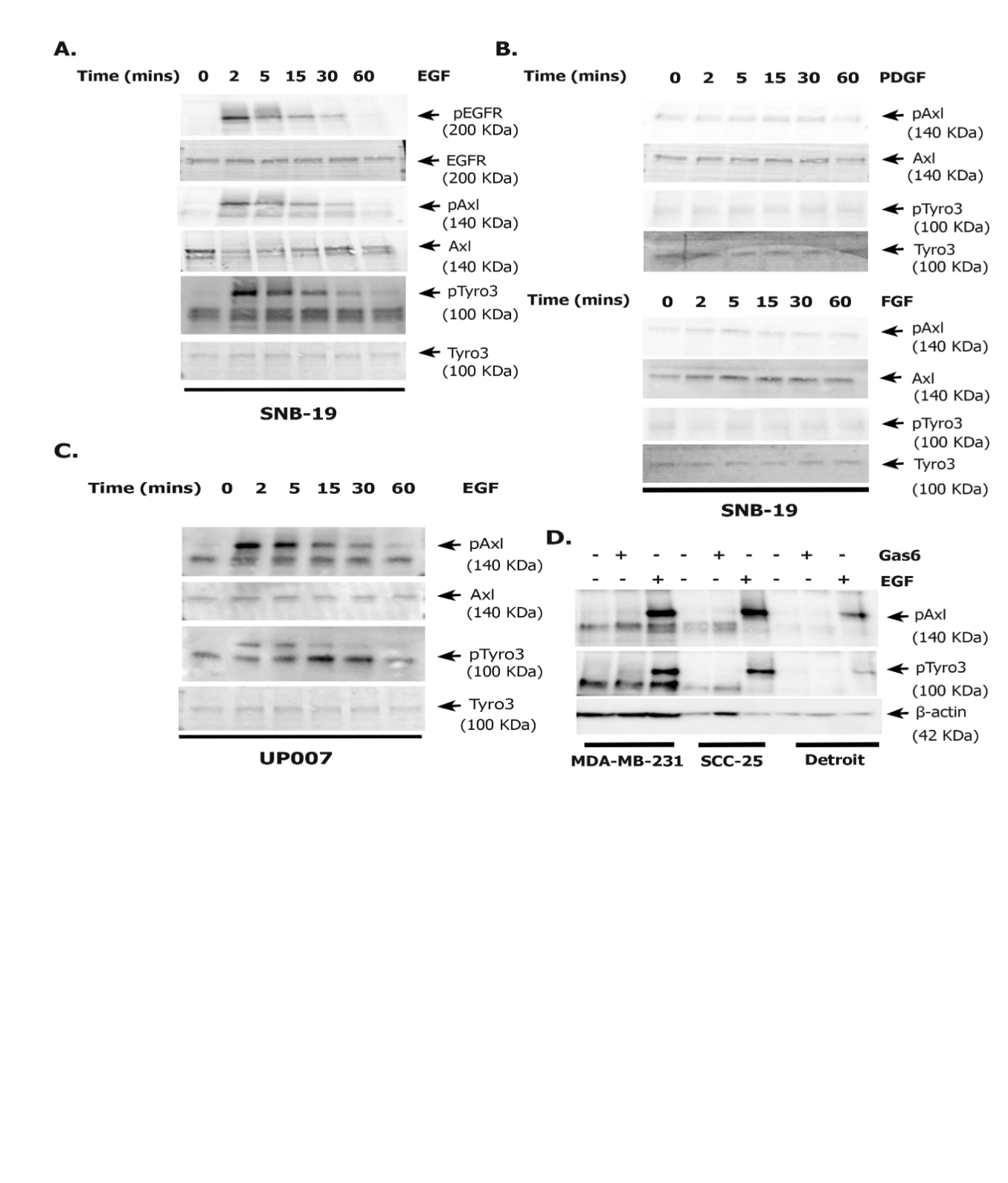


EGF stimulates Axl phosphorylation in a variety of human cancer cell lines. Western blot of phosphorylated Axl (pY779) after 10 min stimulation with Gas6 (400 ng/ml) or EGF (50 ng/ml) in human breast cancer (MDA-MB-231) and head and neck cancer (SCC-25 and Detroit) cells.
